# Takotsubo cardiomyopathy after a dancing session: a case report

**DOI:** 10.1186/1752-1947-5-533

**Published:** 2011-10-31

**Authors:** Mohammed A Kaballo, Abdelazim Yousif, Awadalla M Abdelrazig, Ammar A Ibrahim, Terence G Hennessy

**Affiliations:** 1 Department of Cardiology, Mid-Western Regional Hospital, Ennis, Ireland

## Abstract

**Introduction:**

Stress-induced (Takotsubo) cardiomyopathy is a rare form of cardiomyopathy which presents in a manner similar to that of acute coronary syndrome. This sometimes leads to unnecessary thrombolysis therapy. The pathogenesis of this disease is still poorly understood. We believe that reporting all cases of Takotsubo cardiomyopathy will contribute to a better understanding of this disease. Here, we report a patient who, in the absence of any recent stressful events in her life, developed the disease after a session of dancing.

**Case presentation:**

A 69-year-old Caucasian woman presented with features suggestive of acute coronary syndrome shortly after a session of dancing. Echocardiography and a coronary angiogram showed typical features of Takotsubo cardiomyopathy and our patient was treated accordingly. Eight weeks later, her condition resolved completely and the results of echocardiography were totally normal.

**Conclusions:**

Takotsubo cardiomyopathy, though transient, is a rare and serious condition. Although it is commonly precipitated by stressful life events, these are not necessarily present. Our patient was enjoying one of her hobbies (that is, dancing) when she developed the disease. This case has particular interest in medicine, especially for the specialties of cardiology and emergency medicine. We hope that it will add more information to the literature about this rare condition.

## Introduction

Takotsubo cardiomyopathy (TCM), or stress-induced cardiomyopathy, is a rare disease that has been increasingly reported during the last decade. It mimics ST elevation myocardial infarction (STEMI), and 1.7% to 2.2% of patients with acute coronary syndrome actually have TCM [1,2]. Patients present with chest pain that usually follows a stressful condition (emotional or physical), electrocardiogram (ECG) changes (ST-segment elevation or T-wave inversion or both), and minor elevation of the cardiac markers [3]. The similarity of TCM to STEMI may subject patients presenting within the time frame of thrombolysis to unnecessary administration of thrombolytic agents. Here, we report a case of TCM in a woman who developed the condition after a dancing session. Fortunately, she did not fulfill the criteria for thrombolysis when she presented to our institute.

## Case presentation

**Figure 1:**
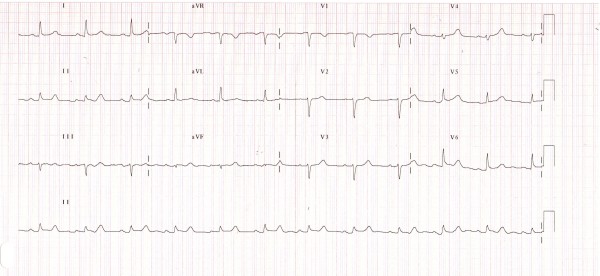
**An electrocardiogram shows minimal ST-segment elevation in leads I and II and aVF, V5, and V6**.

A 69-year-old Caucasian woman with a background of hypercholestrolemia presented with ongoing severe non-radiating central chest pain for the previous 12 hours to our emergency department. Her pain started suddenly 60 minutes after she performed a two-hour dancing session. She had no other symptoms of note. She is an ex-smoker and takes daily aspirin and pravastatin. On presentation, her pulse rate was 60 beats per minute (regular) and her blood pressure was 120/70. The results of a clinical examination were completely normal. An ECG showed ST-segment elevation (not fulfilling the criteria for thrombolysis) in leads I and II and aVF, V5, and V6 (Figure 1).

**Figure 2:**
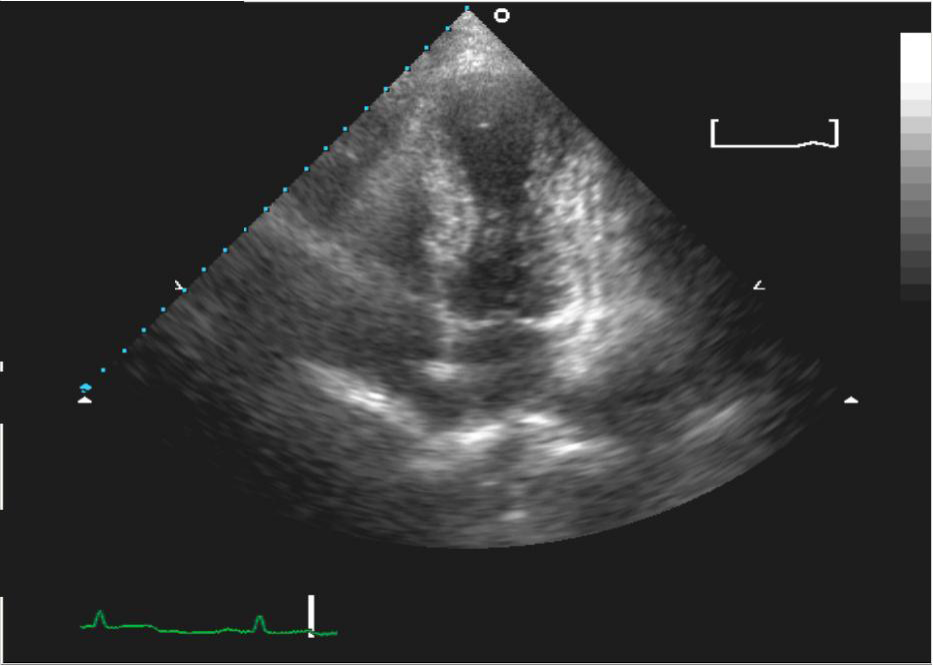
**Echocardiography findings of apical hypokinesia in a four-chamber view**.

**Figure 3:**
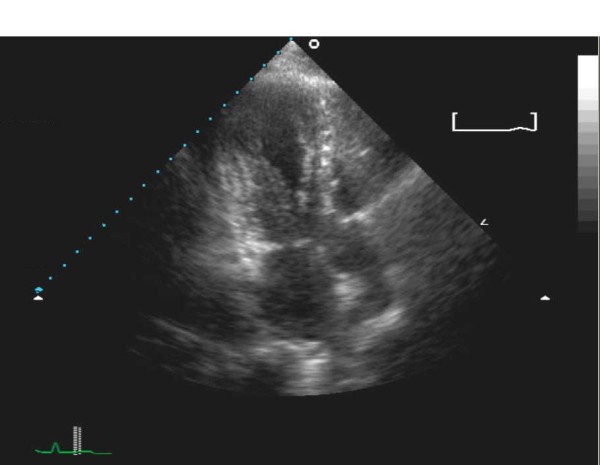
**Echocardiography findings of apical hypokinesia in a two-chamber view**.

Immediately, she was started on a loading dose of aspirin and clopidogrel, given glyceryl trinitrate, and commenced on a full dose of enoxaparin. She was admitted to the coronary care unit overnight. Her troponin-T result came back mildly elevated at 0.53 nmol/L. A transthoracic echocardiogram showed a moderate-size left ventricular apical aneurysm with moderate to severe apical wall hypokinesia (Figure 2 and 3) and no evidence of a mural thrombus. The left ventricular function was mildly to moderately reduced; the ejection fraction was 35% to 45%. The right ventricle and all valves were normal.

**Figure 4:**
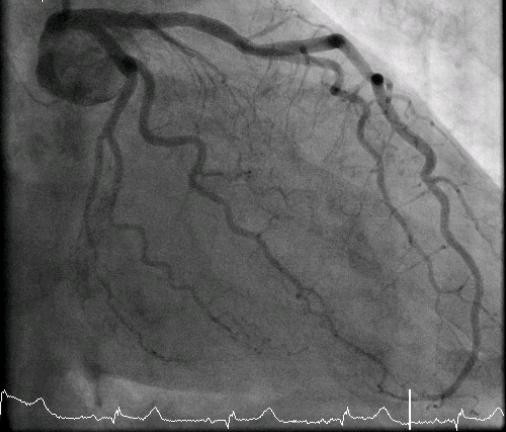
**A left coronary angiogram shows minimal disease**.

**Figure 5:**
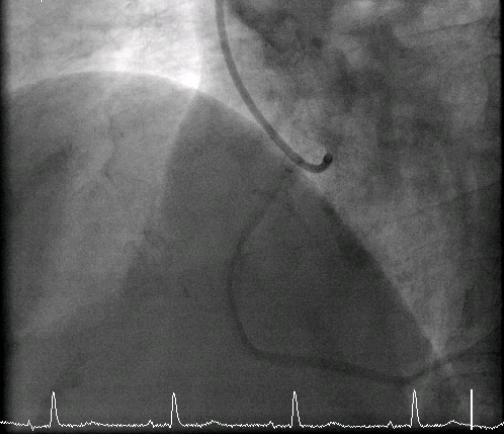
**A normal right coronary angiogram shows minimal disease**.

**Figure 6:**
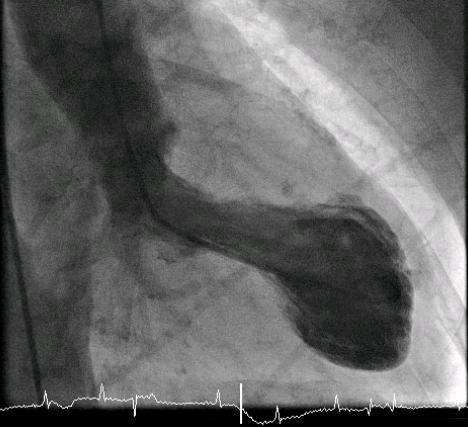
**A ventriculogram shows the typical appearance of the left ventricle during systole**.

**Figure 7:**
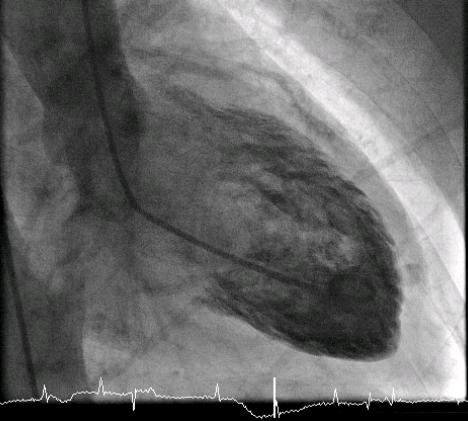
**A ventriculogram shows the typical appearance of the left ventricle during diastole**.

Unfortunately, there are no invasive cardiac services in our hospital and no 24-hour percutaneous coronary intervention (PCI) services in a nearby hospital. So, in the morning (eight hours after presentation), she was transferred to a regional hospital for a coronary angiogram. It showed normal coronary arteries (Figure 4 and 5) and moderate impairment of the left ventricular function (ejection fraction = 30%). The ventriculogram showed end-systole aneurysmal dilatation (ballooning) of both apical and inferior segments of the left ventricle (Figures 5, Figure 6 and 7). A diagnosis of TCM was made.

Investigations for pheochromocytoma were negative, and there was no evidence of myocarditis. She was started on treatment for heart failure-namely ramipril, bisoprolol, and eplerenone-in addition to the medications mentioned above. During her stay in our hospital, she was very stable and did not develop any of the recognized complications of TCM. Ten days later, she was discharged home on the same medications that were started when she was an inpatient. After eight weeks, she was reviewed in the outpatient clinic and a transthoracic echocardiogram was performed. The echocardiogram results were completely normal.

## Discussion

On the basis of the finding of ventricular dysfunction that involved the apex and spared the base of the left ventricle and the absence of stenotic lesions in a coronary angiograph, we believe that our patient had TCM. TCM, or broken heart syndrome, is a type of cardiomyopathy first described in Japan in 1990 by Sato and colleagues [4]. It was named after the Japanese fishing pot used to trap octopus. This disease is characterized by being transient and mimics acute myocardial infarction (MI) in presentation and in ECG findings. It mainly involves postmenopausal women (88.8% of patients) [5]. Patients usually present with chest pain typical of acute coronary syndrome. In most cases, presentation is preceded by an emotionally or physically stressful condition (for example, significant arguments, death of a loved one, natural disasters, legal problems, accidents, surgical procedures, a stay in a critical care unit, and use of illicit drugs) [5]. Stress is absent in one third of patients [6]. Our patient was attending a dancing session the hour before the onset of symptoms, and no preceding emotional stress could be elicited.

A hypothesis by Lyon and colleagues [7] is that TCM is the result of the direct effect of high levels of catecholamines on the ventricular myocardium. High levels of epinephrine are negatively inotropic and switch beta-2-adrenoceptor coupling in ventricular cardiomyocytes from the Gs protein to the Gi protein signaling pathway. The regional nature of the stunning is explained by the presence of more beta-adrenoceptors in the apical myocardium. This effect is reversible after the epinephrine levels return to normal, and this explains why left ventricular function and apical wall motion return to normal within days to weeks of the acute insult [7].

An ECG shows ST-segment elevation or T-wave inversion or both. Dynamic and diffuse T-wave inversions are the most consistent ECG findings in TCM. U waves and long QTc were reported findings [8]. A retrospective case series by Ogura and colleagues demonstrated that a higher ST elevation voltage in leads V4-V6 than in leads V1-V3, in addition to the absence of pathologic Q waves and the absence of reciprocal changes in inferior leads showed high sensitivity and specificity to help differentiate TCM from an anterior MI [9].

Cardiac markers are minimally elevated. When a patient undergoes angiography, no significant coronary artery stenosis is found and left ventricular apical ballooning is present. A variant type, in which only the basal and mid portions of the left ventricle were affected whereas the apex was completely spared, has been described by Karavidas and colleagues [10].

Transthoracic echocardiography is a quick method for viewing wall motion abnormalities typically seen in TCM, specifically hypokinesis or akinesis of the mid and apical segments of the left ventricle.

Treatment of TCM has been that of cardiomyopathy with left ventricular systolic dysfunction-namely, angiotensin-converting enzyme inhibitors, beta-blockers, and diuretics. Calcium channel blockers and aspirin have been recommended. However, none of these medications makes a significant change in outcome or prognosis [11].

In 20% of TCM cases, complications may occur [5]. The following complications have been reported: tachy- and bradyarrhythmias, left ventricular mural thrombus (LVMT), left ventricular outflow obstruction, left heart failure, left ventricular free wall rupture, and cardiogenic shock. Mitral regurgitation has been reported [12]. These complications are life-threatening and should be anticipated in any patient with TCM and appropriate action should be taken as soon as they occur.

The incidence of LVMT in TCM is not well studied. In one study, Haghi and colleagues found LVMT in four out of 52 patients [13]. It can occur both at initial presentation and at anytime later during the disease course. Patients with elevated serum C-reactive protein levels seem to be at higher risk of developing thrombi, as are those with thrombocytosis [13]. Thromboembolic phenomena have been described in three case reports; events included stroke, transient ischemic attacks, and renal infarction [14-16]. According to the literature, the use of anticoagulant therapy administered until complete resolution of wall motion abnormalities appears to be appropriate to treat apical thrombus formation and any possible subsequent embolism [17]. Further anticoagulation with warfarin after recovery of the ejection fraction is not required [18].

The prognosis for TCM is generally excellent; over 90% of patients recover completely, usually within four to eight weeks but sometimes as long as one year [6]. Death may occur in 1% to 3.2% of cases, and the disease may recur in 2% to 3% of cases [19]. Long-term beta-blockers have been shown to reduce recurrences.

As TCM mimics STEMI and patients may present during the time frame of thrombolysis, many of them are subjected to unnecessary thrombolytic therapy, particularly in hospitals with no facilities for immediate coronary angiography. This unnecessary thrombolysis exposes patients to hazards and possible adverse effects but no benefits at all. Also, it appears that thrombolytic therapy is associated with an increased incidence of complications, but there are no controlled studies to support that. Unfortunately, at present, there is no reliable method or criteria to differentiate between TCM with ST elevation and STEMI or to avoid unnecessary thrombolysis. Every effort should be made to study this condition in more depth. We recommend that each and every case be reported to enrich the literature with more information about this uncommon but serious condition. If possible, all patients who had TCM should be followed for many years to look for long-term sequels. Transthoracic echocardiography is an easy-to-use and a non-invasive procedure, and the wall motion abnormalities seen in TCM extend beyond the distribution of any single coronary artery. To avoid unnecessary thrombolysis, we recommend the use of transthoracic echocardiography in the emergency department before thrombolysing postmenopausal women presenting with features of STEMI following emotional stress.

## Conclusions

TCM is an uncommon, though a potentially serious, condition. Although it is commonly precipitated by stressful life events, these are not necessarily present. Our patient was enjoying one of her hobbies (that is, dancing) when she developed the disease. Many patients are thrombolysed unnecessarily because of this condition's close similarity to STEMI. Further reporting and studies in this area are required. To eliminate inadvertent thrombolysis, a well-designed primary PCI or urgent coronary angiography service should be established.

## Abbreviations

ECG: electrocardiogram; LVMT: left ventricular mural thrombus; MI: myocardial infarction; PCI: percutaneous coronary intervention; STEMI: ST elevation myocardial infarction; TCM: Takotsubo cardiomyopathy.

## Consent

Written informed consent was obtained from the patient for publication of this case report and any accompanying images. A copy of the written consent is available for review by the Editor-in-Chief of this journal.

## Competing interests

The authors declare that they have no competing interests.

## Authors' contributions

MAK was the major contributor in studying the case and writing the manuscript and was involved in the medical care of the patient. AAI and AMA were the physicians who admitted the patient, managed her acutely, and assisted with the literature review. AY performed the echocardiography and the coronary angiography. TGH is the head of the department and the consultant cardiologist responsible for the medical care of the patient. All authors read and approved the final manuscript.
